# Calfacilitin is a calcium channel modulator essential for initiation of neural plate development

**DOI:** 10.1038/ncomms2864

**Published:** 2013-05-14

**Authors:** Costis Papanayotou, Irene De Almeida, Ping Liao, Nidia M. M. Oliveira, Song-Qing Lu, Eleni Kougioumtzidou, Lei Zhu, Alex Shaw, Guojun Sheng, Andrea Streit, Dejie Yu, Tuck Wah Soong, Claudio D. Stern

**Affiliations:** 1 Department of Cell and Developmental Biology, University College London, Gower Street (Anatomy Building), London WC1E 6BT, UK; 2 Department of Physiology, Yong Loo Lin School of Medicine, National University of Singapore, 28 Medical Drive, Singapore 117456, Singapore; 3 Research Department, National Neuroscience Institute, Jalan Tan Tock Seng, Singapore 308433, Singapore; 4 Department of Craniofacial Development and Stem Cell Biology, King’s College London, Guy’s Tower Floor 27, London SE1 9RT, UK; 5 These authors contributed equally to this work; 6 Present address: Équipe ‘Specification des destins cellulaires chez la souris’, Institut Jacques Monod, CNRS-Université Paris Diderot, 15 rue Hélène Brion, 75205 Paris, France; 7 Present address: Crystal BioScience, 1450 Rollins Road, Burlingame, California 94010, USA; 8 Present address: Lab for Early Embryogenesis, Riken Center for Developmental Biology, 2-2-3 Minatojima-minamimachi, Chuo-Ku, Kobe 650-0047, Japan

## Abstract

Calcium fluxes have been implicated in the specification of the vertebrate embryonic nervous system for some time, but how these fluxes are regulated and how they relate to the rest of the neural induction cascade is unknown. Here we describe Calfacilitin, a transmembrane calcium channel facilitator that increases calcium flux by generating a larger window current and slowing inactivation of the L-type Ca_V_1.2 channel. Calfacilitin binds to this channel and is co-expressed with it in the embryo. Regulation of intracellular calcium by Calfacilitin is required for expression of the neural plate specifiers *Geminin* and *Sox2* and for neural plate formation. Loss-of-function of Calfacilitin can be rescued by ionomycin, which increases intracellular calcium. Our results elucidate the role of calcium fluxes in early neural development and uncover a new factor in the modulation of calcium signalling.

## Abstract (web summary)

Calcium signalling has been implicated in neural induction in the embryo. The authors identify Calfacilitin as a regulator of CaV1.2 calcium channels, which is required for induction of the pre-neural specifiers Geminin and Sox2 in the early chick embryo.

## Graphical abstract

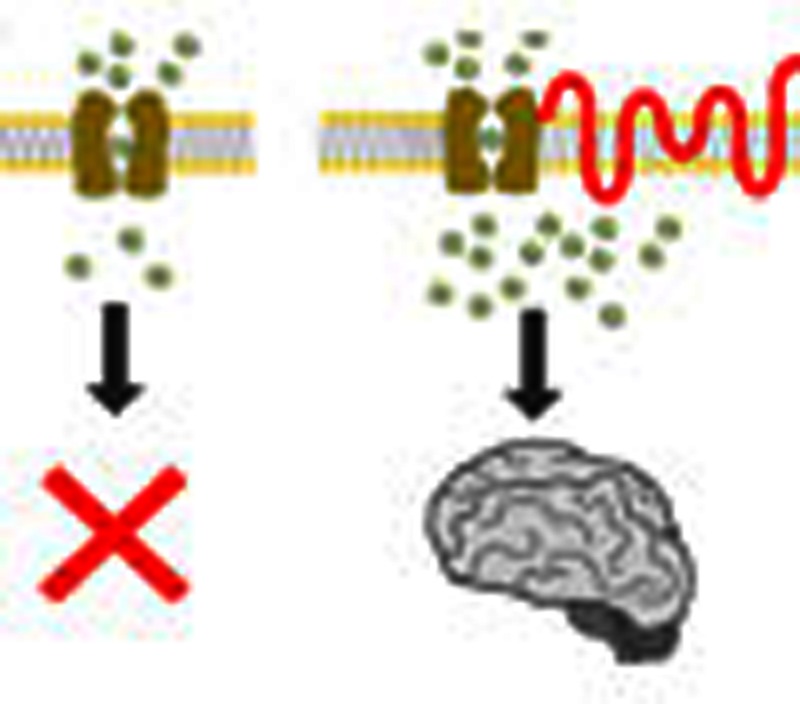

A graft of a special region of the embryo, known as Spemann’s organizer^1^,^2^,^3^ (Hensen’s node in amniotes^4^,^5^,^6^,^7^,^8^,^9^) can trigger the entire process of neural induction in all vertebrate classes, generating a fully patterned central nervous system. For a long time it was thought that a single signal, emitted from the organizer, might account for its neural inducing activity. Consistent with this, a large body of research, mainly in amphibian embryos, suggested that BMP inhibition is a sufficient signal to trigger the entire process of neural induction^1^,^10^,^11^,^12^,^13^,^14^.

However, development of the neural plate takes a relatively long time; for example, in the chick embryo expression of the earliest definitive neural plate marker, *Sox2*, requires about 12 h exposure of competent ectoderm to neural inducing signals from a grafted organizer and a morphologically recognizable neural plate only appears several hours later^15^,^16^,^17^,^18^. Also, experiments in a variety of systems have suggested that signals other than BMP inhibition are required for neural induction, including fibroblast growth factor (FGF)^19^,^20^,^21^,^22^,^23^,^24^, Wnt inhibition^25^,^26^ and calcium/protein kinase-C signalling^27^,^28^,^29^,^30^,^31^,^32^,^33^,^34^,^35^,^36^,^37^. However, how these signals are integrated, from which tissues they emanate and in which order, is not yet understood. FGF signalling is partly integrated with BMP signalling through MAP kinase-dependent phosphorylation of the linker region of the BMP effector Smad1^38^, but FGF inhibition experiments^19^,^20^ suggest that FGF signals are also required independently of BMP inhibition.

Timed organizer transplantation and removal experiments suggested that a minimum of 5 h signalling from a grafted Hensen’s node is required for the responding epiblast to become sensitive to BMP inhibition^39^,^40^,^41^,^42^. To uncover the events that occur during these initial 5 h of neural induction (upstream of BMP inhibition), we conducted a differential screen between chick epiblast cells that had or had not been exposed for 5 h to neural inducing signals from a graft of the organizer, Hensen’s node^21^,^43^,^44^,^45^. Twelve differentially expressed genes were identified, encoding proteins involved in transcriptional regulation (ERNI^17^,^21^, Churchill^43^, Sox3, Otx2^46^), known and putative receptors (TrkC and Asterix^45^), a putative RNA-binding protein (Obelix^45^), the retinoid regulator Cyp26A1^46^ and proteins with pro- and anti-apoptotic functions (Dad1, Fth, HCF^44^). These genes are expressed at characteristic times following a graft of the organizer, suggesting that the organizer induces a hierarchical succession of states (‘epochs’)^45^. In addition to the above genes, one further gene with differential expression, initially designated C3, was identified in the screen. It encodes a protein of unknown function and has not yet been investigated. This is the subject of the present study.

Here we show that C3 encodes a multi-pass transmembrane protein, which facilitates calcium signalling through Ca_V_1.2 L-type channels and therefore named it Calfacilitin. We further show that Calfacilitin is required for neural induction (a requirement that can be bypassed by forcing calcium entry into the responding tissue). Calfacilitin is induced by FGF and is required for induction of *Geminin* and *Sox2*, two key effectors of neural induction. Our findings uncover a new regulator of calcium signalling as well as a new link between calcium and other signals during neural induction.

## Results

### Isolation of Calfacilitin

**Figure 1:**
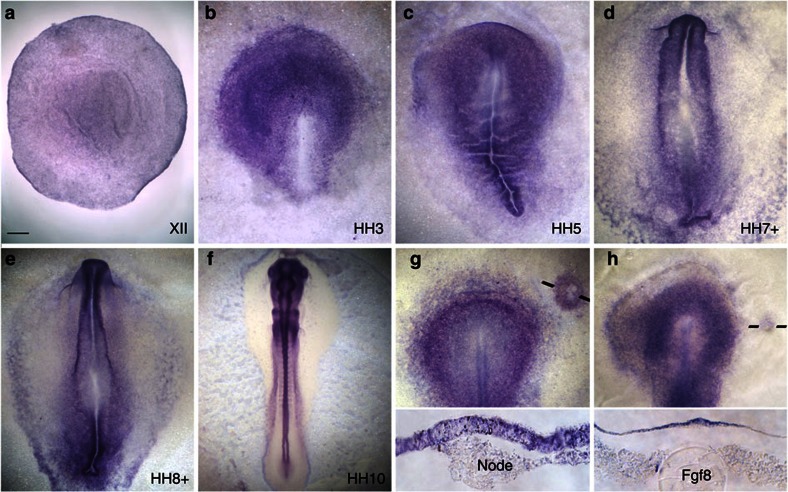
*Calfacilitin* expression and regulation. (**a–f**) Expression in the chick embryo at stages XII-10. Expression gradually becomes restricted to the central nervous system. (**g**, Top) A graft of Hensen’s node induces *Calfacilitin*; in section (**g**, bottom), Calfacilitin induction is seen in the host epiblast. (**h**, Top) FGF8 induces *Calfacilitin*; this is seen more clearly in section (**h**, bottom), which also reveals expression localized to the host epiblast next to the FGF8 bead. Scale bar, 300 μm in **a**; Scale bar, 100 μm in **b**,**c**,**g**,**h**; Scale bar, 120 μm in **d**,**e**; Scale bar, 500 μm in **f**, Scale bar, 60 μm in **g** bottom, **h** bottom.

*In situ* hybridization reveals that expression of *C3* mRNA starts weakly in the epiblast before gastrulation (Fig. 1a), then increases in the future neural plate and thereafter remains expressed throughout the central nervous system (Fig. 1b–f); this pattern of expression is almost identical to the pre-neural marker *Sox3*^47^,^48^. As with *Sox3*, a graft of the organizer upregulates expression of the novel gene within 3 h in competent epiblast (18/20 at 3 h, 8/8 at 5 h; Fig. 1g). This is mimicked by FGF8 (19/26 in 3 h, 13/13 in 5 h; Fig. 1h) but not by other secreted proteins including BMP antagonists (Chordin, Noggin), Cerberus, Dkk1 or Crescent (not shown). Its normal expression and the dynamics of its regulation by organizer signals and by FGF therefore suggest a possible early role for the C3 product in the cascade of events leading to neural plate formation.

**Figure 2:**
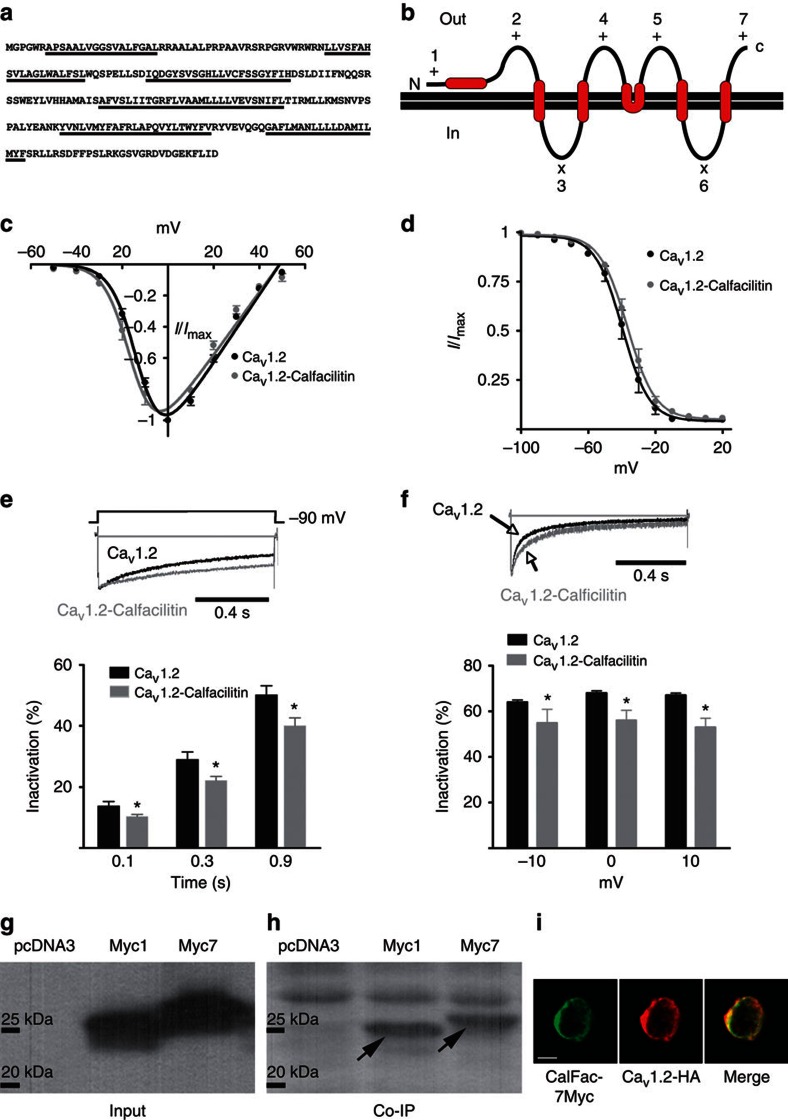
Calfacilitin electrophysiological properties and interactions with calcium channel Ca_V_1.2 Predicted aminoacid sequence (**a**) and membrane topology (predicted transmembrane domains underlined) (**b**) of Calfacilitin. The number on each of the loops corresponds to the position of a Myc epitope in constructs (Calfacilitin-myc1-7; see Supplementary Fig. 1) used for epitope mapping experiments. +: Stains with anti-myc without detergent; x: negative. (**c**) Normalized I–V curve for *I*_Ba_. *V*_0.5_ (Ca_V_1.2)=−12.44±0.5 mV (*n*=5). *V*_0.5_ (Ca_V_1.2-Calfacilitin)=−15.4±0.8 mV (*n*=8). *P*=0.0245 (Student’s *t*-test). (**d**) Steady-state inactivation properties. *V*_0.5_ (Ca_V_1.2)=-39.7±0.8 mV (*n*=6). *V*_0.5_ (Ca_V_1.2-Calfacilitin)=−36.72±0.6 mV (*n*=5). *P*=0.0165 (Student’s *t*-test). (**e**) Representative *I*_Ba_ during depolarizations to *V*_max_ and percentage *I*_Ba_ inactivation. (**f**) Representative *I*_Ca_ and percentage *I*_Ca_ inactivation during depolarizations to −10, 0 and 10 mV at 0.1 s after peak current. *P<0.05 (Student’s *t*-test). Error bars in panels **c**–**f** correspond to the s.e.m. (**g**,**h**) Co-immunoprecipitation experiment demonstrating that Calfacilitin binds to Ca_V_1.2. g shows the input lysate (western blot with anti-myc); (**h)** shows the results of precipitation with anti-Ca_V_1.2 and detection with anti-myc. pcDNA3 was included as a control (lane 1) and the experiment performed with two different myc-tagged versions of Calfacilitin, in positions 1 (lane 2, Myc1) and 7 (lane 3, Myc7) (see b above). (**i**) Co-localization of Calfacilitin (here Myc7 construct; green) with Ca_V_1.2 (HA-tagged; red) in HEK293T cells. Scale bar, 15 μm.

**Figure 3:**
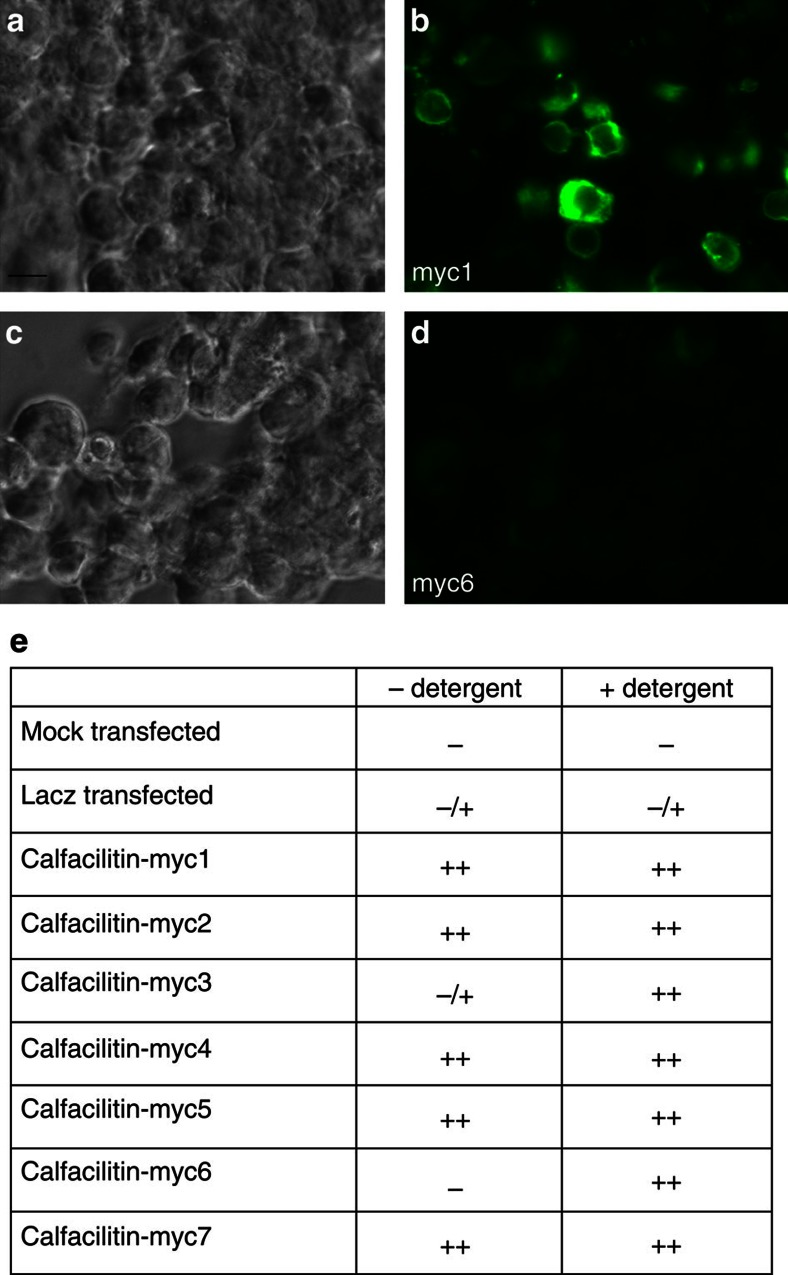
Experiment to elucidate the membrane topology of Calfacilitin. (**a–d**) Examples of results obtained. (**a,b**) HEK293T cells transfected with Calfacilitin-myc1 and stained without detergent, seen by phase contrast (**a**) and fluorescence (**b**). Surface staining is seen. (**c,d**) cells stained with Calfacilitin-myc6 without detergent seen by phase contrast (**c**) and fluorescence (**d**)—no signal is apparent. (**e**) Summary of results of transfections of COS or HEK293T cells with Calfacilitin tagged with a myc epitope in different positions (as shown in Fig. 2b). —denotes no signal, −/+ denotes a very weak signal and ++ denotes strong staining with anti-myc. The deduced topology is shown in Fig. 2b. Scale bar (for **a**–**d**), 30 μm.

Sequence analysis predicts a protein of 252 aminoacids with several putative transmembrane regions (Fig. 2a), containing a TLC (‘TRAM, LAG1, CLN8’) domain, present in many proteins with important roles including lipid synthesis, transport and sensing^49^,^50^,^51^,^52^. This appears to be highly conserved across the vertebrates (Supplementary Fig. S1). To determine the topology of the protein within the membrane, we performed epitope mapping experiments. A myc epitope was introduced into seven different locations between the predicted transmembrane domains, each construct introduced into either COS or HEK293T cells, which were then fixed and stained with anti-Myc either in the presence of detergent (to detect both intracellular and extracellular epitopes) or without detergent (for extracellular epitopes) (Fig. 3). The results suggest a 6-pass transmembrane topology with both the N- and C-termini outside the cell (Fig. 2b). This topology is reminiscent of some ion channels, including Ca^2+^ channels^53^, which have been implicated in early neural development in Xenopus^29^,^30^,^31^,^33^,^34^,^36^,^37^,^54^,^55^.

### Calfacilitin is a facilitator of Ca_V_1.2 channels

**Figure 4:**
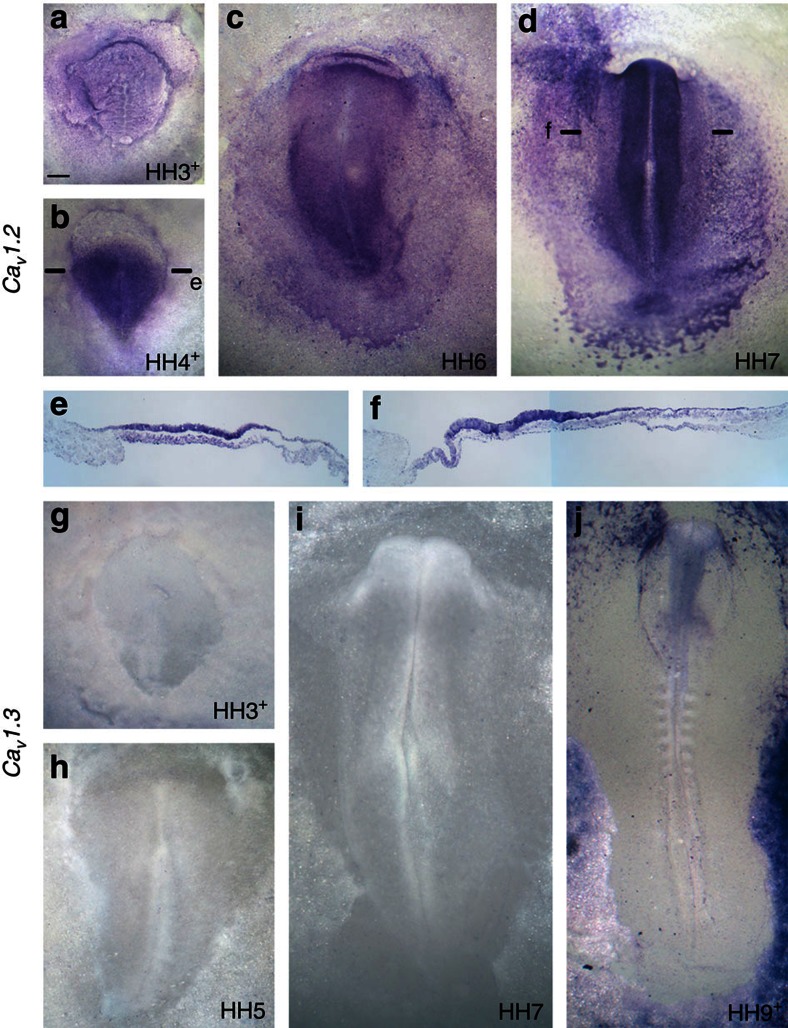
Expression of the L-type calcium channels Ca_V_1.2 and Ca_V_1.3 in normal embryos. (**a–f**) Ca_V_1.2. Expression gradually becomes concentrated to neural plate precursors. (**a**) Mid-primitive streak stage (HH3+); (**b**) late primitive streak stage (HH4+); (**c**) early neurulation (HH6); (**d**) neural plate stage (HH7). (**e,f**) Sections through the levels indicated in (**b**,**d)**, showing expression mainly in the epiblast. (**g–j**) In contrast, no significant expression of the Ca_V_1.3 channel in seen in any embryonic region at primitive streak (**g,h**), neurulation (**i**) or neural tube (**j**) stages. Scale bar, 200 μm (**a**,**b**,**g**); Scale bar, 120 μm (**c**–**f**,**h**); Scale bar, 90 μm (**i**); Scale bar, 230 μm (**j**).

These observations raised the question of whether the C3 protein functions as an ion channel. We transfected the cDNA into HEK293T cells but detected no currents in whole-cell patch clamp electrophysiological recordings. However, when co-transfected with L-type Ca_V_1.2 calcium channels, I-V relationships were shifted towards a negative potential (Fig. 2c; *P*<0.025, Student’s *t*-test) and the steady-state inactivation towards a positive potential (Fig. 2d; *P*<0.02, Student’s *t*-test), therefore evoking a larger window current. Furthermore, C3 slows inactivation in the presence of either Ba^2+^ or Ca^2+^ as the charge carrier (Fig. 2e). To test its specificity for Ca_V_1.2 channels, we examined its effects on another major L-type channel, Ca_V_1.3, and found no significant effect (Supplementary Fig. S2). To determine whether these channels are expressed appropriately in the embryo, we performed *in situ* hybridization: *Ca*_*V*_*1.2* mRNA is expressed in the prospective neural plate with a pattern and dynamics of expression very similar to those of the novel protein (Fig. 4a–f), whereas *Ca*_*V*_*1.3* expression is not detected before neural tube formation (Fig. 4g–j).

Does the protein interact directly with Ca_V_1.2? To test this we performed co-immunoprecipitation (IP) experiments in HEK293T cells. Two tagged versions (with a Myc-tag at position 1 or 7 in Fig. 2b) can be co-precipitated with Ca_V_1.2 (Fig. 2g). Moreover, immunostaining for Myc-tagged-C3 and Ca_V_1.2 reveals co-localization of both at the cell membrane (Fig. 2i). Together, these data suggest that although the novel protein is not itself an ion channel, it enhances calcium influx into the cell through the L-type calcium-channel Ca_V_1.2, to which it can bind within the cell membrane and with which it is co-expressed in the embryo. We therefore named this protein Calfacilitin.

### Neural induction requires *Ca* ^*2+*^ signals

**Figure 5:**
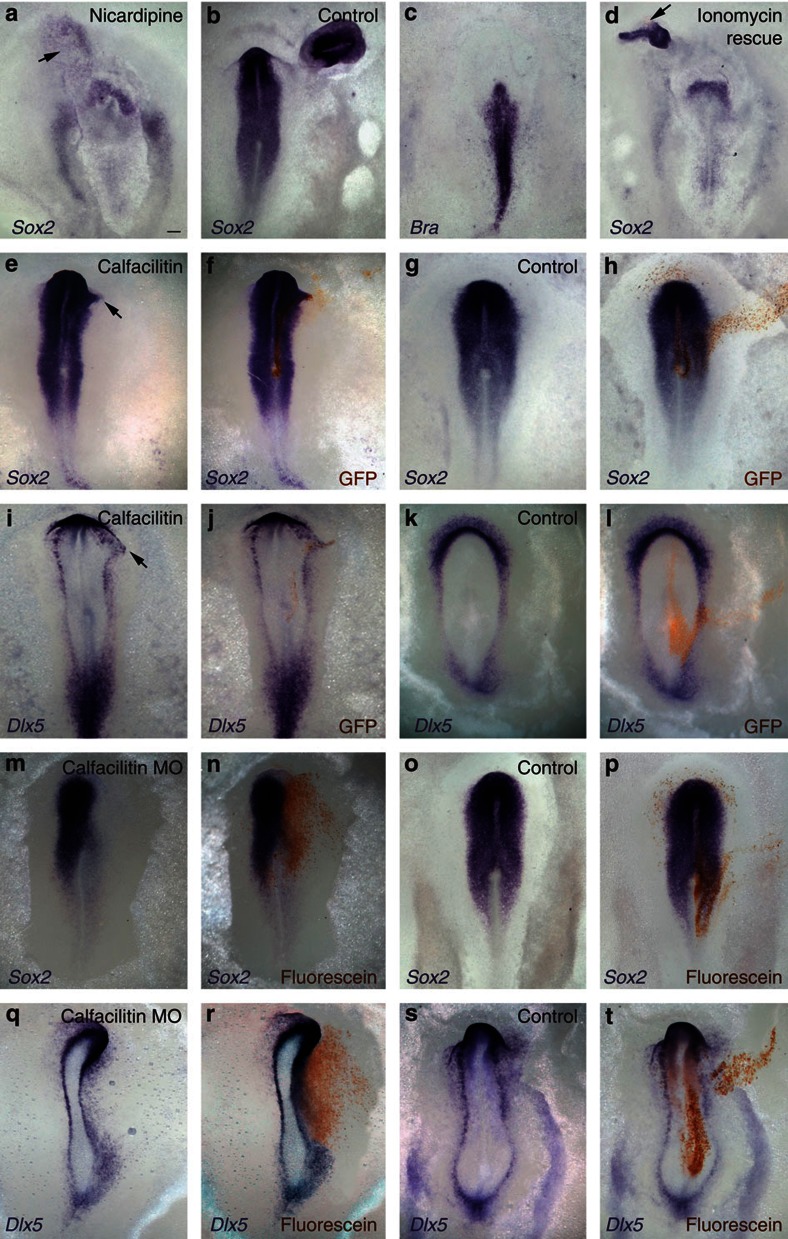
Calfacilitin is required for neural plate specification. (**a–d**) Nicardipine inhibits neural plate development and neural induction by a grafted node (arrow) (**a**,**b**: DMSO control); (**c**) this treatment does not affect mesoderm (expression of *Brachyury* shown). The effect of nicardipine can be rescued by an ionomycin-soaked bead (**d**, arrow). (**e–l**) Gain-of-function: Calfacilitin (**e**,**f**,**i,j**) but not control GFP (**g,h**,**k,l**) expands the neural plate, revealed by the neural plate marker *Sox2* (**e**–**h**) and the border marker *Dlx5* (**i**–**l**). (**i**–**t**) Loss-of-function: Calfacilitin MOs (**m,n**,**q,r**) unlike control MOs (**o,p**,**s,t**), reduce the neural plate, as seen by expression of the neural plate marker *Sox2* (**m**–**p**) and the border marker *Dlx5* (**q**–**t**). *In situ* probe (purple) indicated on the lower left of each panel. Anti-GFP (brown in **f,h,j,l**) or anti-fluorescein (for MO; brown in **n,p,r,t**) reveal electroporated cells. Scale bar, 100 μm (for all panels).

As L-type calcium channels have been implicated in neural induction in Xenopus^31^,^33^,^34^,^36^,^54^, we tested whether this is also the case in the chick by exposing embryos to nicardipine, a selective blocker of L-type calcium channels, before grafting Hensen’s node from a donor embryo. No expression of the neural plate marker *Sox2* was observed either in the host neural plate (whose development was also severely impaired; Fig. 5a) or in the epiblast adjacent to the grafted organizer (6/20 with expression; Fig. 5a, arrow), unlike controls (15/16 expressing; Fig. 5b). Nicardipine did not affect markers of non-neural tissue including *Brachyury* (7/7 expressing; Fig. 5c) and *Chordin* (6/6; Supplementary Fig. S3). The abnormal development of the host could be due to non-specific toxicity, but *Sox2* induction by the node can be rescued in nicardipine treated embryos with beads soaked in the calcium ionophore ionomycin, which drives Ca^2+^ ions into the cell independent of any channels (16/19 expressing; Fig. 5d, arrow). Dramatically, induction can be rescued even when the host neural plate is severely affected (Fig. 5d). These results demonstrate that induction of the neural plate by the organizer requires Ca^2+^ influx, normally through nicardipine-sensitive, L-type calcium channels.

To test this further we used various Ca^2+^ indicators that fluoresce in proportion to the free intracellular Ca^2+^ concentration (Rhod-2, Fura2 or Fura-Red with Fluo4). Whole embryos present particular challenges to Ca2+-imaging, which are further complicated by combining in experiments using fluorescein-labelled morpholinos and GFP-labelled reporter constructs (see below). The main problem is imaging large areas of the embryo with enough sensitivity and discrimination of Ca^2+^ levels over a long period of time (hours) without loss of dye from the cells. We explored Fura2 (ratio-imaged with IR excitation in a multi-photon microscope or with UV excitation by standard fluorescence microscopy), the ratio between Fura-Red+Fluo4 in a confocal or conventional fluorescence microscope, and Rhod-2 (conventional fluorescence). The latter was most stable and allowed combining with FITC-labelled morpholino or GFP-labelled constructs. Although Rhod-2 tends to accumulate in mitochondria, the emitted signal is still sensitive to cytosolic Ca2+ levels; for example, this can be seen in ionomycin control experiments (see below). With the microscopy methods used, in whole embryos, we were unable to detect fast Ca^2+^ transients either in the normal embryo or in experimentally manipulated regions. However, we were able to detect slow changes over several hours.

**Figure 6:**
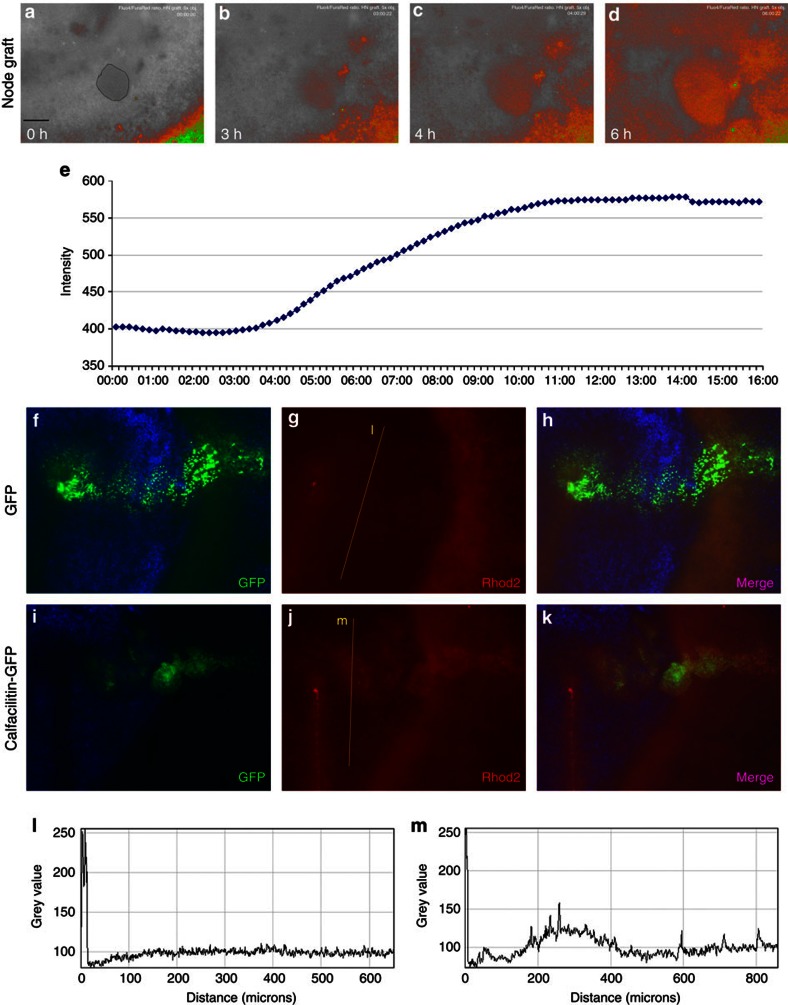
Both the organizer (Hensen’s node) and Calfacilitin increase intracellular calcium. (**a**–**e**) The organizer induces an increase in intracellular calcium in responding cells starting about 4 h after grafting. This example shows the ratio between green and red emissions of Fluo4/Fura-Red excited at 488 nm 0, 3, 4 and 6 h after grafting (**a**–**d**; pseudo-colour encoded as a heat map). Panel **e** shows a time-scan of the changes in the green channel (Fluo4 signal) in the region of the graft. The site of the grafted node is outlined by a thin line in **a**. (**f**–**m**) Embryo loaded with the calcium indicator Rhod-2 after electroporation with GFP alone (**f**–**h**) or Calfacilitin+GFP (**i**–**k**), imaged 6 h after electroporation. Calfacilitin increases the calcium signal as compared with GFP alone. Scans showing the relative intensity of the Rhod-2 signal in **g,j** at the position indicated by the line are shown in (**l**,**m**), respectively, (the scan line was positioned parallel to the axis of the primitive streak, ~150 μm lateral to the midline). Scale bar (**a**–**d**,**f**–**k**), 100 μm.

Organizer grafts into embryos loaded with these dyes cause an increase in the Ca^2+^ signal, starting after about 3–4 h in the adjacent host epiblast (Fig. 6a–e). Interestingly, this is also the time required for the node to induce expression of *Calfacilitin* in host epiblast. We also tested whether Calfacilitin is sufficient to cause a rise in intracellular Ca^2+^ *in vivo* by electroporating dye-loaded embryos with an expression plasmid driving either Calfacilitin-IRES-GFP or just GFP (Calfacilitin-IRES-RFP or DS-Red in some experiments). Calfacilitin-transfected cells display elevated Ca^2+^ as compared with neighbouring non-transfected cells or to control GFP- or DS-Red-transfected cells (Fig. 6f–m). Therefore, both misexpression of Calfacilitin and neural induction by the organizer are accompanied by a rise in intracellular Ca^2+^.

### Calfacilitin and *Ca* ^*2+*^ are not sufficient for neural induction

To determine whether Calfacilitin is sufficient to induce *Sox2* in competent epiblast, we electroporated Calfacilitin-IRES-GFP as a line extending from the prospective neural plate to the lateral, non-neural epiblast. This construct (26/32; Fig. 5e; arrow), but not GFP alone (0/11; Fig. 5g), expands *Sox2* expression into the embryonic non-neural epiblast. When these embryos are examined by *in situ* hybridization for the neural plate border marker *Dlx5*, this border is found shifted towards the non-neural ectoderm (Calfacilitin: 6/10 shifted, Fig. 5i, arrow; Control: 0/6 shifted, Fig. 5k). However, neither Calfacilitin nor ionomycin beads induce *Sox2* in the more peripheral area opaca epiblast (not shown). These effects resemble the activity of BMP antagonists, which can only expand the neural plate and its border into the adjacent non-neural ectoderm when misexpressed along a continuous line of cells extending from the neural plate^40^,^42^,^56^. These observations suggest that neither Calfacilitin nor an increase in intracellular Ca^2+^ is sufficient for neural induction, which involves additional signals.

### Calfacilitin is required for neural induction

**Figure 7:**
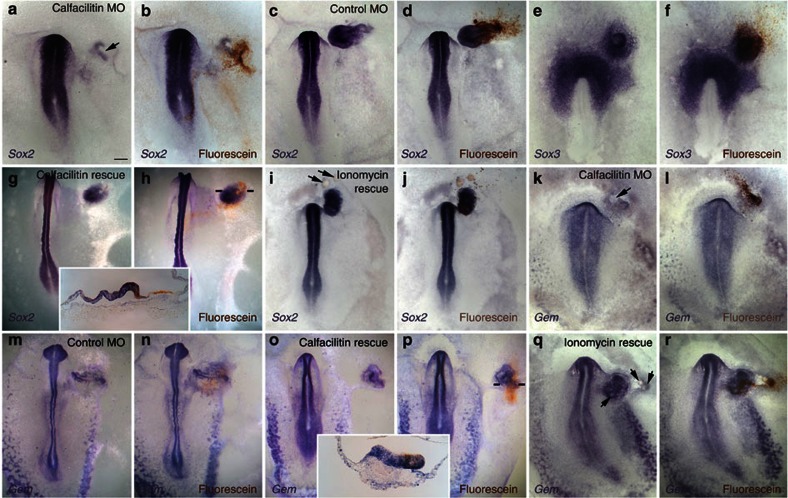
Calfacilitin is required for neural induction. Calfacilitin MOs (**a**,**b**) but not control MOs (**c,d**) block induction of *Sox2* by Hensen’s node, but do not affect induction of the earlier marker *Sox3* (**e,f**). The effect can be rescued both by Calfacilitin cDNA (**g,h** top, bottom shows a section revealing *Sox2* rescue in the host epiblast) and by the Calcium ionophore ionomycin (**i,j**). Hensen’s node grafts have reduced ability to induce *Geminin* in Calfacilitin-MO-electroporated epiblast (**k,l**) as compared with control-MO epiblast (**m,n**). This induction can also be rescued either by co-electroporation of Calfacilitin cDNA (**o**,**p** top, bottom shows a section revealing *Geminin* rescue in the host epiblast) or by ionomycin beads (**q,r**). *In situ* probe (purple) indicated on the lower left of each panel. Anti-fluorescein (to reveal fluorescein-labelled MO; brown in **b**,**d**,**f**,**h** top, **j**,**l**,**n**,**p**,**r**) reveal electroporated cells. Scale bar, 100 μm (**a**,**b**,**e**,**f**,**h** bottom,**k**,**l**); Scale bar, 180 μm (**c**,**d**,**i**,**j**,**o**–**r**); Scale bar, 400 μm (**g**,**h** top, **m**,**n**); Scale bar, 50 μm (**p** bottom).

To establish whether Calfacilitin is required for neural plate development, we electroporated morpholino oligonucleotides (MO) targeting internal splice sites of *Calfacilitin* into the prospective neural plate of early primitive streak stage embryos. When electroporated into the embryo, this causes exon skipping, generating a truncated Calfacilitin protein (Supplementary Fig. S4). First, we measured the effect of the knockdown on intracellular Ca^2+^ levels in the prospective neural plate. Control-MO electoporated cells showed no significant difference to neighbouring non-electroporated tissue (ratio: 1.02; two-tailed *t*-test *P*=0.25; *n*=12), whereas Calfacilitin-MO significantly lowers Ca^2+^ with respect to neighbouring cells (1.6-fold reduction; two-tailed *t*-test *P*=2.32 × 10^−5^, *n*=12; in these experiments, rectangles of 224 × 170 pixels in the electroporated area were measured). Next, we determined whether this treatment affects expression of the neural plate marker *Sox2*. Indeed it does (17/22 affected; Fig. 5m), unlike control-MO (0/9; Fig. 5o). To test the fate of the cells that had lost *Sox2* expression we examined the border marker *Dlx5*: this was found to shift into the neural plate, expanding the non-neural ectoderm territory (Calfacilitin-MO: 5/6 shifted, Fig. 5q ; Control-MO: 0/6, Fig. 5s). We also tested whether neural induction by a grafted organizer requires Calfacilitin: we electroporated MOs into competent epiblast and then grafted a node onto the electroporated site. Calfacilitin-MO completely blocked *Sox2* induction in 21/31 embryos and substantially reduced it in a further 6/31 (Fig. 7a), as compared with control-MO (22/26 with normal expression; Fig. 7c). Calfacilitin-MO did not affect the expression of earlier pre-neural markers (*Sox3* and *ERNI*; 19/22; Fig. 7e). These results show that Calfacilitin is required for neural induction, acting downstream of *Sox3* and *ERNI* but upstream of *Sox2*.

**Figure 8:**
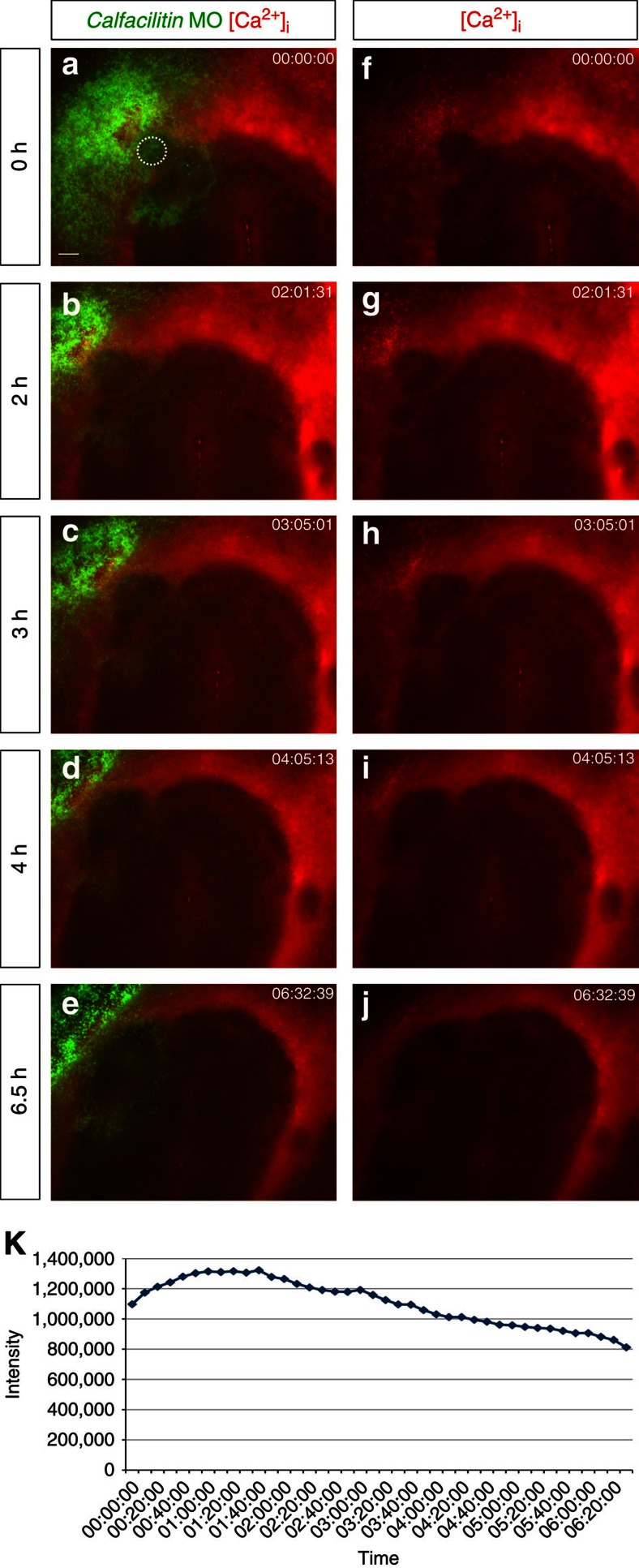
Calfacilitin-MO lowers intracellular calcium in epiblast adjacent to a grafted Hensen’s node. Embryos were loaded with the Ca^2+^ indicator Rhod-2 and electroporated with Calfacilitin MOs before grafting an organizer (Hensen’s node) onto the electroporated region (stippled circle at 0 h). An embryo is shown 0 (**a**,**f**), 2 (**b**,**g**), 3 (**c**,**h**), 4 (**d**,**i**) and 6.5 (**e**,**j**) hours after the graft, revealing progressive loss of calcium signal under the graft. The left panels show the Ca^2+^ signal (red) overlapped with the fluorescein (green) revealing the MO; the right panels reveal only the Ca^2+^ signal. (**k**) Quantification of the Ca^2+^ signal in the MO-electroporated epiblast adjacent to the node graft of the same embryo (measured in the area outlined by a circle in a, comprising 13,996 pixels) over time. Compare with Fig. 6e for node graft without MO-electroporation). Scale bar (**a**–**j**), 100 μm.

As a further test of the specificity of the Calfacilitin-MO we tested whether the phenotype can be rescued with *Calfacilitin* cDNA (lacking the targeted splice site). This is the case (36/42 embryos with rescued *Sox2* expression; Fig. 7g). As a more dramatic test of the importance of Calfacilitin in relation to its calcium-regulating functions, we explored whether the effect of the MO can be rescued by ionomycin. When a node is grafted onto Calfacilitin-MO-transfected epiblast along with ionomycin-soaked beads, *Sox2* expression is restored (14/21; Fig. 7i). This is not due to induction of *Sox2* by ionomycin, as no ectopic expression is seen when ionomycin beads are grafted alone (see above). This predicts that the MO lowers Ca^2+^ in cells adjacent to the grafted node; to test this, we imaged Ca^2+^ using Rhod-2. The MO prevented the increase in Ca^2+^ caused by node grafts (see above and Fig. 6) and also reduced the Ca^2+^ signal with respect to neighbouring regions of the host within 3–4 h, with continued decline over at least 6 h (Fig. 8). Together, these results strongly suggest that Calfacilitin is necessary for neural induction, through its role in Ca^2+^ signalling.

### Calfacilitin regulates Geminin expression

Calcium channels have been proposed to be required for the expression of *Geminin* in Xenopus^54^. As Geminin was recently implicated in the acquisition of neural fate and initiation of *Sox2* expression in chick and mouse^17^, we tested whether Calfacilitin can induce *Geminin*. Although Calfacilitin alone is not sufficient to induce *Geminin* in competent area opaca epiblast (not shown), Calfacilitin-MO (3/20 with expression; Fig. 7k), but not control-MO (8/8; Fig. 7m), inhibits upregulation of *Geminin* by a node graft. This effect can be rescued by *Calfacilitin* cDNA lacking splice sites (39/43; Fig. 7o) as well as by ionomycin beads (7/10; Fig. 7q). Together, these results suggest that induction of *Geminin* is downstream of Calfacilitin.

## Discussion

Our results uncover Calfacilitin as a previously undiscovered regulator of Ca^2+^ signalling and as a new factor in neural induction. It is required early, downstream of initial FGF signals and defines an essential step for the later acquisition of *Sox2* expression and neural plate formation. We demonstrate that its action in these processes is through modulation of intracellular Ca^2+^ levels. As FGF induces expression of both *Geminin*^17^ and *Calfacilitin*, this could explain the finding that FGF can activate calcium channels in Xenopus ectoderm. A possible mechanism might involve arachidonic acid and TRPC channels^37^, similar to the lipid-regulating properties displayed by other members of the TLC family^49^,^50^,^51^. This could represent the long-sought mechanism for how L-type calcium channels are involved in neural induction.

## Methods

### Embryo manipulations and in situ hybridization

Brown Bovan Gold strain chick eggs (obtained from Henry Stewart & Co., UK) were used for most experiments. Quail eggs were obtained from Potter Poultry Farm, UK. All animal experiments conform to UK Home office regulations and were restricted to embryos during the first 2 days of incubation. Embryos were staged according to Hamburger and Hamilton^57^ for primitive streak and later stages and following Eyal-Giladi and Kochav^58^ for earlier stages. They were explanted and cultured by the method of New^59^ but using a ring of square cross-section and the embryo grown in a Petri dish^60^. Node grafts^4^ were performed using chick or quail donor nodes and chick host embryos. FGF8 was administered by soaking heparin-acrylic beads (Sigma) in FGF8 (R&D) diluted in Pannett–Compton saline, briefly washed in the same saline and implanted into the desired region of the host^17^,^21^,^43^. *In situ* hybridization was performed using DIG-labelled cRNA probes and detected using NBT-BCIP^61^.

### Calcium imaging

To visualize Ca^2+^ levels, embryos were incubated in Rhod-2 (Invitrogen R-1245MP, 0.5 μg ml^−1^ in 0.1% DMSO in Pannett–Compton saline), or a mixture of Fura-Red and Fluo4 (Invitrogen; each at 2 μg ml^−1^ in 0.4% DMSO and 0.08% pluronic in Pannett–Compton saline) or Fura2 (Invitrogen; 2 μg ml^−1^ in DMSO/Pluronic as above) at 37 °C for 30 min, rinsed in saline and left to recover for 30 min–1 h in new culture before grafting a node and/or electroporation of either Control-MO or Calfacilitin-MO. Time-lapse imaging was performed (image frame size=1,344 pixels × 1,024 pixels). Fura2 was imaged in a conventional fluorescence microscope as the ratio between emission at 510 nm generated by excitations at 340 and 380 nm or in a confocal microscope by excitation at 351 and 364 nm. Fluo4 and Fura-Red were excited at 488 nm and the ratio between green and red fluorescence generated was imaged by conventional microscopy. Rhod-2 was imaged by conventional fluorescence, using TRITC filters.

For quantification in Rhod-2 experiments with electroporation of a morpholino or GFP construct, Ca^2+^ levels (red signal) were measured in fixed areas (a rectangle or circle) of electroporated and contralateral control tissue, measuring the total intensity of red pixels; Student’s *t*-tests were used for analysis. In Ca^2+^-imaging experiments, implantation of a AG1X2 bead soaked in 2 μM ionomycin (Sigma I9657) in DMSO caused an explosive increase in the calcium signal with all dyes—this was performed as a control in time-lapse calcium imaging experiments. For inhibition of calcium channels, embryos were incubated in nicardipine (Sigma N7510, 100 μg ml^−1^ in 0.1% DMSO in Pannett–Compton saline) at 37 °C for 30 min and rinsed in saline before incubation of the embryo. To drive Ca^2+^ into cells, AG1X2 beads were incubated in 2 μM ionomycin in DMSO, rinsed in Pannett–Compton and implanted into embryos.

### Misexpression of Calfacilitin

A fragment of *Calfacilitin* was identified from a previously described screen^21^ and the full-length cDNA isolated as described^43^. For gain-of-function experiments, the *Calfacilitin* ORF was cloned into pCAβ-IRES-GFP and electroporated from a stock at 0.5 mg ml^−1^. To facilitate visualization, GFP cloned in the same vector was co-electroporated. For loss-of-function, fluorescein-labelled morpholinos (Gene Tools) (GTAGCCTGCAATGTAAGAGAAGAGC, CTCCCCTACAGCCGCACTCACCATG) targeting intron–exon splice sites of Calfacilitin were electroporated either singly or together, from a stock containing 0.5 mM of each. The efficiency of the MO was tested by RT–PCR using primers: CalFac-F 5′-GAACCTCCTCGTTTCCTTCG-3′ and CalFac-R 5′-ACGAGGCCAACAAGTACGTC-3′. Glyceraldehyde-3-phosphate dehydrogenase (GAPDH) was used as a loading control, with primers: GAPDH-F 5′-GTGGGGGAGACAGAAGGGAAC-3′ and GAPDH-R (5′-AGAGGTGCTGCCCAGAACATC-3′ (Supplementary Fig. S3).

### Molecular characterization of Calfacilitin

To study the topology of the transmembrane domains of Calfacilitin, a myc-tag was introduced after aminoacids 3, 42, 74, 107, 163, 201 and 252 of Calfacilitin to produce seven different tagged versions (with a myc epitope in the N-terminus, in one of the five loops between the predicted transmembrane domains or in the C-terminus of the protein) and cloned into pcDNA3.1. The constructs were transfected into COS cells using Lipofectamine-2000^40^ or into HEK293T cells using PEI^62^ and the cells obsserved by fluorescence microscopy.

Haemagglutinin (HA) tagged Ca_V_1.2 was a gift from Dr Emmanuel Bourinet to TWS and contains the HA epitope in the extracellular S5-H5 loop of domain II^63^. HA-Cav1.2 (2.6 μg), β2a (2 μg), Myc-Calfacilitin (2.3 μg) and TAG (0.6 μg) were transfected into HEK cells with lipofectamine. After 48 h, the cells were fixed with 4% paraformaldehyde and stained using mouse-Anti-HA (Cat. No. 11 666 606 001, Roche) and rabbit-Anti-c-MYC (C3956, Sigma), both at 1:100. The secondary antibodies (1:400 dilution) were Alexa Fluor 594 chicken anti-mouse IgG (H+L) (A21201, Invitrogen) and Alexa Fluor 488 donkey anti-rabbit IgG (H+L) (A21206, Invitrogen). No detergent was used in this procedure. The images were visualized using a confocal microscope (Fluoview BX61; Olympus).

### Electrophysiology

The electrophysiological properties of Calfacilitin were studied by patch clamp techniques after transfection into HEK293 using calcium phosphate^64^. For IP experiments, HEK293 cells were transfected with Ca_V_1.2 α1, β_2a_-GFP, α_2_δ-subunits, TAG and C3-myc using lipofectamine 2000(Invitrogen). Control cells were transfected with an equal amount of pcDNA3 vector. 24 h later, the cells were harvested and lysed in 100 μl Hepes lysis buffer containing: 20 mM Hepes, 137 mM NaCl, 1% Triton X-100, 10% glycerol, 1.5 mM MgCl_2_ and 1 mM EGTA. After incubation for 1 h at 4 ^°^C, 50 μl of protein lysate were taken as input and the remaining for IP. Eight microgram anti-Ca_V_1.2 antibody (Alomone ACC-003) were incubated with the protein lysate and protein A beads (Roche) for 3 h. After washing three times with lysis buffer containing 0.1% Triton X-100, the protein was separated by SDS–PAGE. Calfacilitin-myc was detected with anti-myc antibody (Sigma C3956) and Ca_V_1.2 protein by anti-Ca_V_1.2 antibody.

## Author contributions

C.P. and I.D.A. performed all the embryo experiments, some of them with participation of E.K., L.Z. and A.Sh. P.L. performed the electrophysiological experiments in T.W.S.’s laboratory with the assistance of S.-Q.L. and D.Y. G.S. and A.S. performed the initial screen that led to isolation of Calfacilitin. N.M.M.O. performed transfection experiments in COS cells. The project was conceived and directed by C.D.S. and largely performed in his laboratory. The paper was written by C.D.S. with contributions from C.P., I.D.A., P.L. and T.W.S.

## Additional information

**Accession codes**: Sequence data have been deposited in Genbank/EMBL/DDBJ under accession number GQ504719.

**How to cite this article:** Papanayotou, C. *et al.* Calfacilitin is a calcium channel modulator essential for initiation of neural plate development. *Nat. Commun.* 4:1837 doi: 10.1038/ncomms2864 (2013).

## Supplementary Material

Supplementary Information Supplementary Figures S1-S4
