# Supplementary material for: Calfacilitin is a calcium channel modulator essential for initiation of neural plate development
Source: Nat Commun. 2013 May 14;4:1837–. doi: 10.1038/ncomms2864 (PMC3674269; doi:10.1038/ncomms2864)
Supplement: Supplementary Information — Supplementary Figures S1-S4 [file ncomms2864-s1.pdf]

Supplementary Fig. S1. **Calfacilitin is conserved among different species**.

```
Calfacilitin       ---MGPGWRAPSAALVGGSVALFGALRRAALALPRPAAVRSRPGRVWRWRNLLVSFAHSV
Human              ----MPRLLHPALPLLLGATLTFRALRRALCRLPLPVHVRADPLRTWRWHNLLVSFAHSI
Chimpanzee         ----MPRLLHPALPLLLGATLTFRALRRALCRLPLPVHVRADPLRTWRWHNLLVSFAHSI
Mouse              ----MPLLFHPAWPLLLGATLTFRALRRVLCRLPQPAHVQTDPLRTWRWHNLLVSFTHSI
Zebrafish          MDTWLNEVQKFPVLYVLCCSVLFRILHWCLQIVARPDTVTKDRWKTWKWRNLSVSLVHSL
                              .   :  .   *  *:     :. *  *     :.*:*:** **:.**:

Calfacilitin       LAGLWALFSLWQSPELLSDIQDGYSVSGHLLVCFSSGYFIHDSLDIIFNQQSRSSWEYLV
Human              VSGIWALLCVWQTPDMLVEIETAWSLSGYLLVCFSAGYFIHDTVDIVASGQTRASWEYLV
Chimpanzee         VSGIWALLCVWQTPDMLVEIETAWSLSGYLLVCFSAGYFIHDTVDIVTSGQTRASWEYLV
Mouse              VSGIWALLCLWQTPEMLVEIETAWSASGYLLVCFSAGYFIHDTVDIVVSKQTRASWEYLV
Zebrafish          LTGTWAVACVIYYPAMVHEIHSTYTPSAYMLVVVSSGYFIEDAADIVFSGHAKASWEFLL
                   ::* **: .:   * :: :*.  :: *.::** .*:****.*: **: . ::::***:*:

Calfacilitin       HHAMAISAFVSLIITGRFLVAAMLLLLVEVSNIFLTIRMLLKMS-NVPSPALYEANKYVN
Human              HHVMAMGAFFSGIFWSSFVGGGVLTLLVEVSNIFLTIRMMMKIS-NAQDHLLYRVNKYVN
Chimpanzee         HHVMAMGAFFSGIFWSSFVGGGVLTLLVEVSNIFLTIRMMMKIS-NAQDHLLYRVNKYVN
Mouse              HHVMAMGAFFSGIFWKRFVGGGVLTLLVEVSNIFLTLRMMMKIN-NAQDLLLYKVNKYIN
Zebrafish          HHVLVLWCFLYAVFTHQYVAGAVVALFVEVNSVFLHTRLLLNLAKVAHSSLIYTVNKVLN
                   **.:.: .*.  ::   :: ..:: *:***..:**  *:::::   . .  :* .** :*

Calfacilitin       LVMYFAFRLAPQVYLTWYFVRYVEVQGQGAFLMANLLLLDAMILMYFSRLLRSDFFPSLR
Human              LVMYFLFRLAPQAYLTHFFLRYVNQRTLGTFLLGILLMLDVMIIIYFSRLLRSDFCP---
Chimpanzee         LVMYFLFRLAPQAYLTHFFLRYVNQRTLGTFLLGILLMLDVMIIIYFSRLLRSDFCP---
Mouse              LVMYFLFRLAPQAYLTKFFLQYAGQRTLGTFLLAILLMLDLMIIIYFSRLLRSDFCP---
Zebrafish          VVTYVTFRLGAQFYLTWYLTYHYSSLDYALYFLITTMLMNIMILIYFYRLIRSDFFTKRR
                   :* *. ***..* *** ::  :      . :::   :::: **::** **:**** .

Calfacilitin       KGSVGRDVDGEKFLID
Human              -EHVPKKQHKDKFLTE
Chimpanzee         -EHVPKKQHKDKFLTE
Mouse              -ERAPRRQQKDKFLTE
Zebrafish          IQNGIQKLAAD-----
                        :    :
```

Sequence alignment of the predicted protein sequence of Calfacilitin with its putative orthologues in other species. Chick Calfacilitin has been submitted to GenBank and assigned accession number GQ504719. Orthologues: Zebrafish: AAH83250.1; Mouse: NP_080984; Chimpanzee: XP_511362.1; Human: BC014072.1. Shaded areas correspond to the predicted transmembrane domains, which are conserved.

Supplementary Fig. S2. Lack of effect of calfacilitin on $Ca_V1.3$ channels.

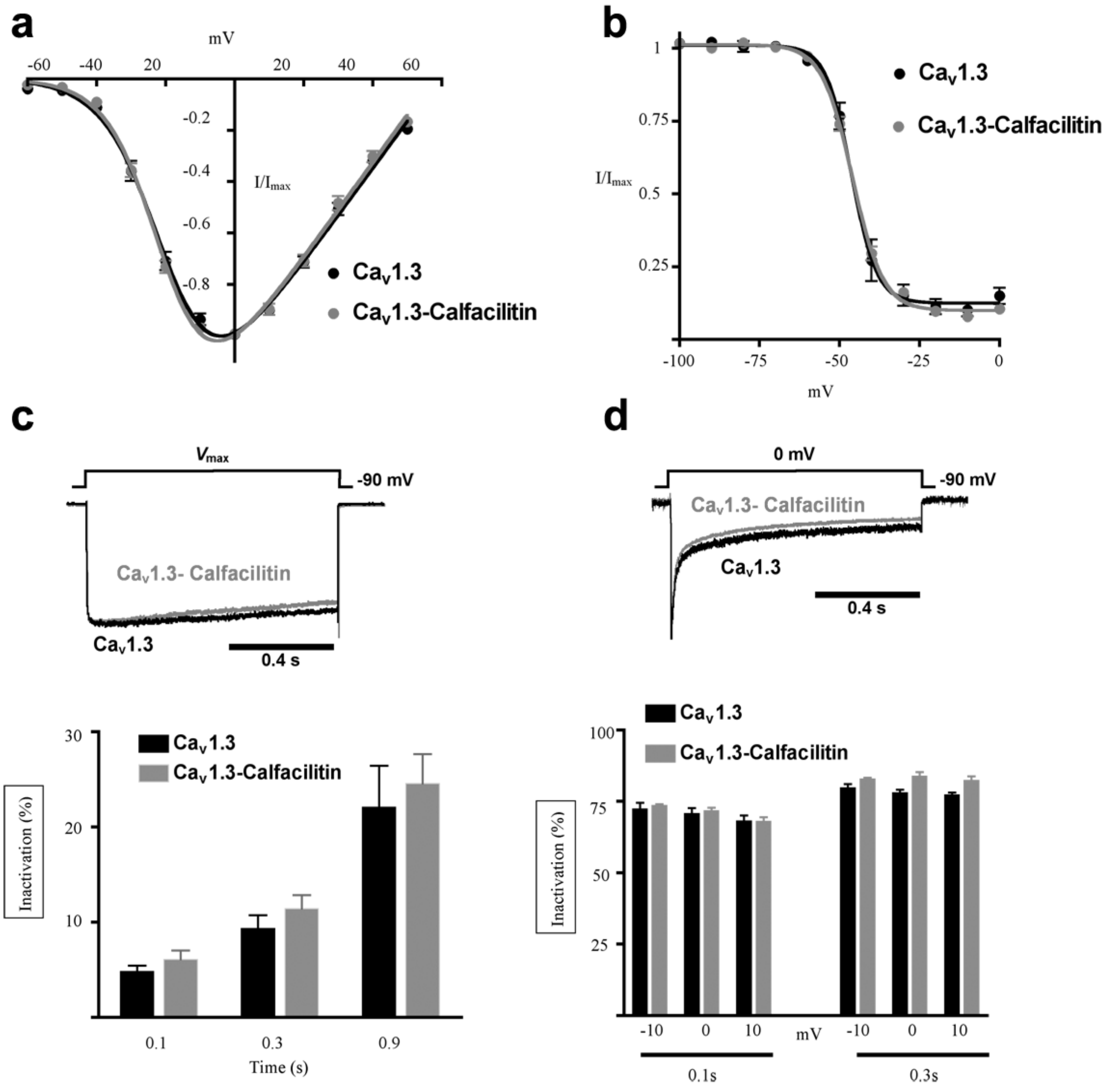

a. Normalized *I-V* curved for I_{Ca}. $V_{0.5}$ ($Ca_V1.3$) = -19.36±0.64 mV (n=7). $V_{0.5}$ ($Ca_V1.3$-Calfacilitin) = -20.0±0.53 mV (n=5). P>0.05 (Student's *t*-test). **b.** Steady-state inactivation properties. $V_{0.5}$ ($Ca_V1.3$) = -46.3±0.64 mV (n=7). $V_{0.5}$ ($Ca_V1.3$-Calfacilitin) = -45.9±0.3 mV (n=7). P>0.05 (Student's *t*- test). **c.** Representative I_{Ba} during depolarizations to V_{max} and percentage of I_{Ba} inactivation. No difference was found between the presence and absence of calfacilitin. N_{cell}=7 ($Ca_V1.3$). N_{cell}=5 ($Ca_V1.3$-Calfacilitin). **d.** Representative I_{Ca} and percentage I_{Ca} inactivation during depolarizations to -10, 0 and 10 mV at 0.1 and 0.3 s after peak current. There is no difference between the presence and absence of calfacilitin. N_{cell}=7 ($Ca_V1.3$). N_{cell}=7 ($Ca_V1.3$-Calfacilitin). Error bars in all panels correspond to the standard error of the mean.

Supplementary Fig. S3. Nicardipine treatment does not affect expression of *Chordin*.

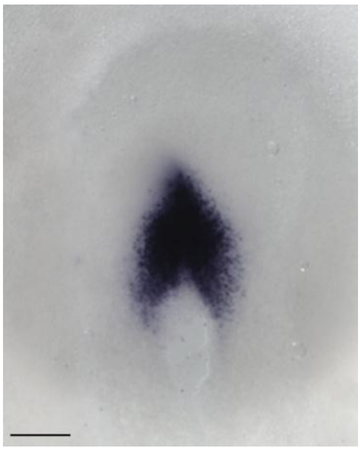

Embryo treated with Nicardipine as shown in Fig. 5c, after in situ hybridisation with the organizer marker *Chordin*. This embryo is at stage 4^+ and expression is confined, as in normal embryos, to the organizer and emerging head process (dark blue signal). Scale bar: 100µm.

Supplementary Fig. S4. Morpholino directed against an exon-intron junction of Calfacilitin causes exon-skipping and generates a truncated form.

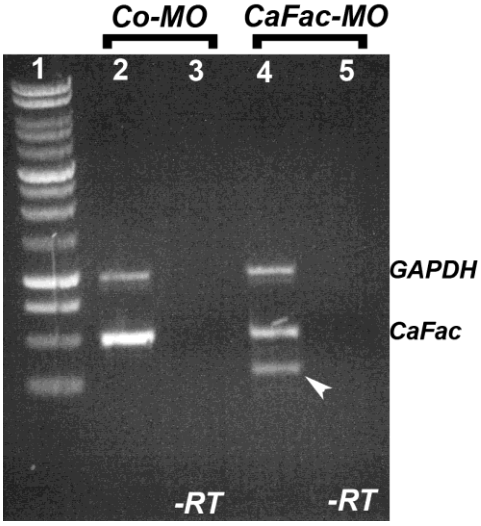

Embryos were electroporated with Control (Co-MO, Lanes 2-3) or Calfacilitin (CaFac-MO, Lanes 4-5) morpholinos, the latter targeting a splice junction. The products were amplified by RT-PCR. Lane 1, size markers (Promega 1kb ladder; the lowest four markers correspond to 253, 500, 750 and 1000 bp respectively, from bottom to top). Lanes 3 and 5 contain the same samples as 2 and 3 but Reverse Transcriptase (RT) was omitted as a control. Glyceraldehyde-3-Phosphate Dehydrogenase (GAPDH) was used as a loading control. The Morpholino causes a decrease in the Calfacilitn (CaFac) band and appearance of a smaller band corresponding to a truncated version caused by exon skipping (arrowhead). Note that electroporation targets a mosaic of cells therefore each sample is a mixture of targeted and normal cells; this can account for the remaining full-length Calfacilitin in the CaFac-MO sample.
